# lncRNA ENSRNOT00000087717 mediated differentiation of satellite glial cells derived from dorsal root ganglion via AKT1

**DOI:** 10.1590/1414-431X2025e14265

**Published:** 2025-10-06

**Authors:** Jinwei Yang, Sijia Zhang, Xiaohua Weng, Chunyan Li, Li Shen, Yun Shang, Wei Ma, Liyan Li

**Affiliations:** 1 Institute of Neuroscience, Kunming Medical University, Kunming, Yunnan, China; 2 Second Department of General Surgery, First People's Hospital of Yunnan Province, Kunming, Yunnan, China; 3 Department of Neurology, Second Affiliated Hospital of Kunming Medical University, Kunming, Yunnan, China

**Keywords:** DRG, SCGs, Differentiation, Lnc87717, AKT1

## Abstract

Satellite glial cells (SGCs) within the dorsal root ganglion (DRG) possess the potential for transdifferentiation. Our previous study (doi: 10.1007/s12257-019-0317-x) has identified lncRNA ENSRNOT00000087717 (lnc87717) and its target AKT1 as potential regulators in the differentiation process of DRG-SGCs. In this study, the cell morphology of SGCs was assessed using immunofluorescence cytochemistry during the differentiation following knockdown of lnc87717, as well as downregulation or upregulation AKT1. The subcellular localization of lnc87717 was visualized by fluorescence *in situ* hybridization (FISH). The mRNA expression levels of lnc87717, AKT1, BDNF, TrkB, proBDNF, and p75^NTR^ were assessed using qRT-PCR. For the *in vivo* study, 27 male Sprague-Dawley rats aged 6-9 weeks were used to establish the sciatic nerve injury model. The number of apoptotic cells in DRGs was subsequently detected in the AKT1 inhibitor, activator, and control group after the administration of proBDNF antiserum. *In vitro*, following knockdown of lnc87717, there was a significant decrease in the expression levels of proBDNF, BDNF, TrkB, and p75^NTR^ (P<0.05). Furthermore, the number of nestin-positive SGCs and the expression of lnc87717 and AKT1 were increased in the AKT1-activated group (P<0.05). *In vivo*, compared to the control group, the number of apoptotic cells in the DRG was increased in the AKT1-inhibited group. Additionally, the expressions of lnc87717 and AKT1 were significantly upregulated (P<0.05), whereas the expression levels of PI3K, NF-κB, and Bad were significantly downregulated (P<0.05) in DRGs following AKT1 up-regulation compared to those in the control group. The differentiation of DRG-SGCs is suggested to be mediated through the activation of AKT1, while lnc87717 downregulates AKT1.

## Introduction

Sensory neuron bodies encased by satellite glial cells (SGCs) within dorsal root ganglia (DRGs) can release neurotransmitters such as glutamate, adenosine triphosphate, and substance P and express various receptors for neurotransmitters and inflammatory mediators (1,2). SGCs play crucial roles beyond merely supporting, protecting, and nourishing neurons; they express diverse cell markers reflecting their heterogeneity (3,4). These neuroglial cells actively regulate physiological pain and undergo morphological changes during neural inflammation (5-7). It has been discovered that neurogenesis, or the increase in the number of neurons, also occurs in the central nervous system (CNS) and peripheral nervous system (PNS), particularly in adult DRG and trigeminal ganglia (8-11). Neural stem cells were not identified in the PNS ganglia (12,13). Previous studies demonstrated that SGCs in the adult DRG function as precursors of sensory neurons during postnatal development and following nerve injury. *In vitro* culture of SGCs derived from neonatal rat DRGs can induce differentiation into neuronal-like morphology under specific conditions (14). Furthermore, our previous study found an upregulation in the overall expression levels of lncRNA ENSRNOT00000087717 (lnc87717) and its target gene protein kinase B (AKT1) during the differentiation of SGCs into the neuronal-like cells by Kyoto Encyclopedia of Genes and Genomes (KEGG) enrichment and qRT-PCR analyis (15). In the present study, we investigated the potential effect of lnc87717 and its target AKT1 on the differentiation of DRG-SGCs both *in vivo* and *in vitro*.

## Material and Methods

### Animals and grouping

Neonatal male Sprague-Dawley (SD) rats (aged 0 to 24 h) and 6- to 9-week male SD rats weighing 190±10 g were obtained from the Animal Department of Kunming Medical University, Kunming City, China [permit SYXK (Dian) K2020-0006]. This investigation adhered strictly to the Kunming Medical University's guidelines for animal care and use. The Kunming Medical University's Committee on the Ethics of Animal Experiments approved all animal experimental protocols (approval no. KMMU20221575). The flow diagram of the *in vitro* and *in vivo* experiments are shown in Supplementary Figure S1.

For the *in vitro* experiments, a total of 60 neonatal SD rats were used to establish primary DRG-derived-SGCs (DRG-SGCs) cultures. These were allocated into five groups as follows: control DRG-SGCs (DRG-SGC subcultures for 3 days without anti-proBDNF), anti-proBDNF24h (DRG-SGCs treated with anti-proBDNF for 24 h), anti-proBDNF3d (DRG-SGCs treated with anti-proBDNF for 3 days), anti-proBDNF7d (DRG-SGCs treated with anti-proBDNF for 7 days), and anti-proBDNF14d (DRG-SGCs treated with anti-proBDNF for 14 days). Each of these five groups (n=6 rats/group) was subjected to immunofluorescence and qRT-PCR analysis. For experiments aimed at downregulating lnc87717, cells were divided into three groups: anti-proBDNF3d (DRG-SGCs treated with anti-proBDNF for 3 days), NC (cells transfected with a negative control vector for 3 days), and LV-lncRNAi (cells transfected with lnc87717 knockdown lentivirus for 3 days). Each group consisted of five rats. For experiments investigating the effect of AKT1, cells were divided into three groups (n=5 rats/group): anti-proBDNF3d, SC79 (DRG-SGCs treated with anti-proBDNF and the AKT1 activator SC79 for 3 days), and GSK690693 (DRG-SGCs treated with anti-proBDNF and the AKT1 inhibitor GSK690693 for 3 days).

For *in vivo* experiments, a total of 27 adult (6- to 9-week) male SD rats were used for quantitative real-time polymerase chain reaction (qRT-PCR) analysis (control, SC79, and GSK690693, with 9 rats/group).

### Primary culture of SGCs derived from DRGs

Neonatal SD rats were utilized to establish primary SGC cultures from L4-L6 DRGs. The procedure for isolating DRGs and culturing DRG-derived SGCs was as follows: neonatal rats were first sanitized with 75% ethanol and then decapitated. The skin, muscles, and ribs were carefully removed to expose the spine. Using ophthalmic scissors, cuts were made on both sides of the spinous processes. The spinal cord was then extracted, allowing for the extraction of the DRGs from the foramina under a stereomicroscope followed by careful removal of residual nerve fibers. SGCs were harvested from DRG explants without protease digestion and cultured in a humidified 5% CO_2_ incubator (Thermo Fisher Scientific, USA) at 37°C. The cells migrating from the DRGs were subsequently subcultured in SGC medium. Ten days post-subculturing, the cells were transferred to 24-well plates for immunocytochemistry analysis.

### Immunocytochemistry of the cultured cells

To identify the cell types migrating from the DRGs, cells from the five groups were processed and stained for glutamine synthetase (GS), S-100 protein subunit beta (S100β), glial fibrillary acidic protein (GFAP), and nestin. The procedure involved washing the cells thrice with 0.01 mol/L phosphate-buffered saline (PBS) for 30 min, followed by fixation in 4% paraformaldehyde for 20 min at room temperature. Cells were then blocked with 5% donkey serum for 1 h at room temperature before overnight incubation at 4°C with specific primary antibodies (Supplementary Table S1). After primary antibody incubation, cells were washed thrice in PBS with Tween 20 (PBST) and incubated with corresponding fluorescently-labeled secondary antibodies (Supplementary Table S1) for 2 h at room temperature. Following a final wash in PBST, cells were mounted on slides and sealed with nail polish. Fluorescence images were captured using an inverted fluorescence microscope (Leica, Germany) after 3, 7, and 10 days of culture. The absence of immunolabeling in control cells verified the specificity of the antibodies used.

### qRT-PCR

Total RNA from the cells in the five groups (n=5) was isolated using TRI Reagent (Invitrogen, USA). cDNA was synthesized using the RevertAid First Strand cDNA Synthesis Kit (Fermentas, Canada). The mRNA levels were quantified by real-time PCR, using the primers detailed in Supplementary Table S2 and the QuantiTect SYBR Green PCR Master Mix (Qiagen, Germany). Each reaction, with a total volume of 25 µL, included SYBR Green PCR Master Mix, 10 pmol of each primer, and 1 µL of cDNA. A standard dilution series of a sample containing the target gene was used for calibration. The thermocycling conditions were set as follows: initial denaturation at 95°C for 3 min, followed by 40 cycles of 95°C for 15 s, annealing at the primer-specific temperature for 30 s, and extension at 60°C for 40 s. Melting curve analysis from 65 to 95°C was conducted post-amplification to verify the specificity and identity of the PCR products as per the manufacturer's guidelines. Gene expression was calculated using the 2^-ΔΔCT^ method, normalized to GAPDH expression.

### Fluorescence *in situ* hybridization (FISH)

For FISH, cells were first fixed in a 4% paraformaldehyde solution, permeabilized with 0.3% Triton X-100, and subjected to prehybridization. Subsequently, labeled probes were applied and incubated at 37°C overnight in darkness. A FISH kit from RiboBio (China) was used according to the manufacturer's protocol, and images were captured with a Leica confocal microscope. HuR and YAP probes were synthesized by Sangon Biotech (China), with the following sequences: HuR: 5'CY5-CUCUGAGCUCGGGCGAGCAUACGACACCUUA-3'CY5; YAP: 5'FITC-UCUCCGAGUCCCCGCGGACGAGUCACGAUCUGAU-3'FITC.

### Sciatic nerve injury (SNI) model and AKT1 activation or inhibition

Twenty-seven adult SD rats were used to establish the SNI model by unilaterally transection of the left sciatic nerve. Rats with SNI were randomly divided into three groups (9 rats/group): control (rats with SNI receiving intraperitoneal injections of anti-proBDNF for 3 days), SC79 3d (rats with SNI treated with intraperitoneal injections of anti-proBDNF and an AKT1 activator for 3 days), GSK690693 3d (rats with SNI treated with intraperitoneal injections of anti-proBDNF and an AKT1 inhibitor for 3 days). Rats in the control group received intraperitoneal injections of anti-proBDNF serum at a dose of 1 mg/kg on the second day following SNI, twice a week (14). For the administration of AKT1 inhibitor or activator, a dose of 30 mg/kg daily was administered intraperitoneally on the second day after SNI. The treatment was given 5 days per week for a total duration of two weeks (16,17). The rats in each group were euthanized by intraperitoneal injection of excessive pentobarbital sodium at predetermined time points. Afterward, the DRGs from the L4-L6 segments on the injured side were carefully isolated and extracted using dissecting microscopes (Leica, Germany). The DRG tissues for TUNEL assay were immersed in 4% paraformaldehyde and 30% sucrose solution for fixation, and the fresh DRG tissues was then used for qRT-PCR analysis.

### TdT-mediated dUTP nick end labeling (TUNEL)

Apoptotic cells were identified using a fluorescein-labeled TUNEL assay kit. Cells were fixed with 4% paraformaldehyde for 20 min. The cell nuclei were counterstained with 4',6-diamidino-2-phenylindole (DAPI). Imaging was performed using an ABX53 fluorescence microscope (Olympus, Japan). The percentage of TUNEL-positive cells relative to DAPI-positive cells was calculated using ImageJ software (NIH, USA).

### Statistical analysis

The qRT-PCR data from each group and the count of GS and nestin double-labeled positive cells and apoptotic cells were analyzed using one-way analysis of variance (ANOVA) followed by the Tukey *post hoc* test with SPSS 22.0 statistical software for Windows (IBM, USA). The data were normally distributed and homogeneous. Data are reported as means±SEM. A value of P<0.05 or P<0.01 was considered statistically significant.

## Results

### Morphological characteristics of DRG-SGCs

**Figure 1:**
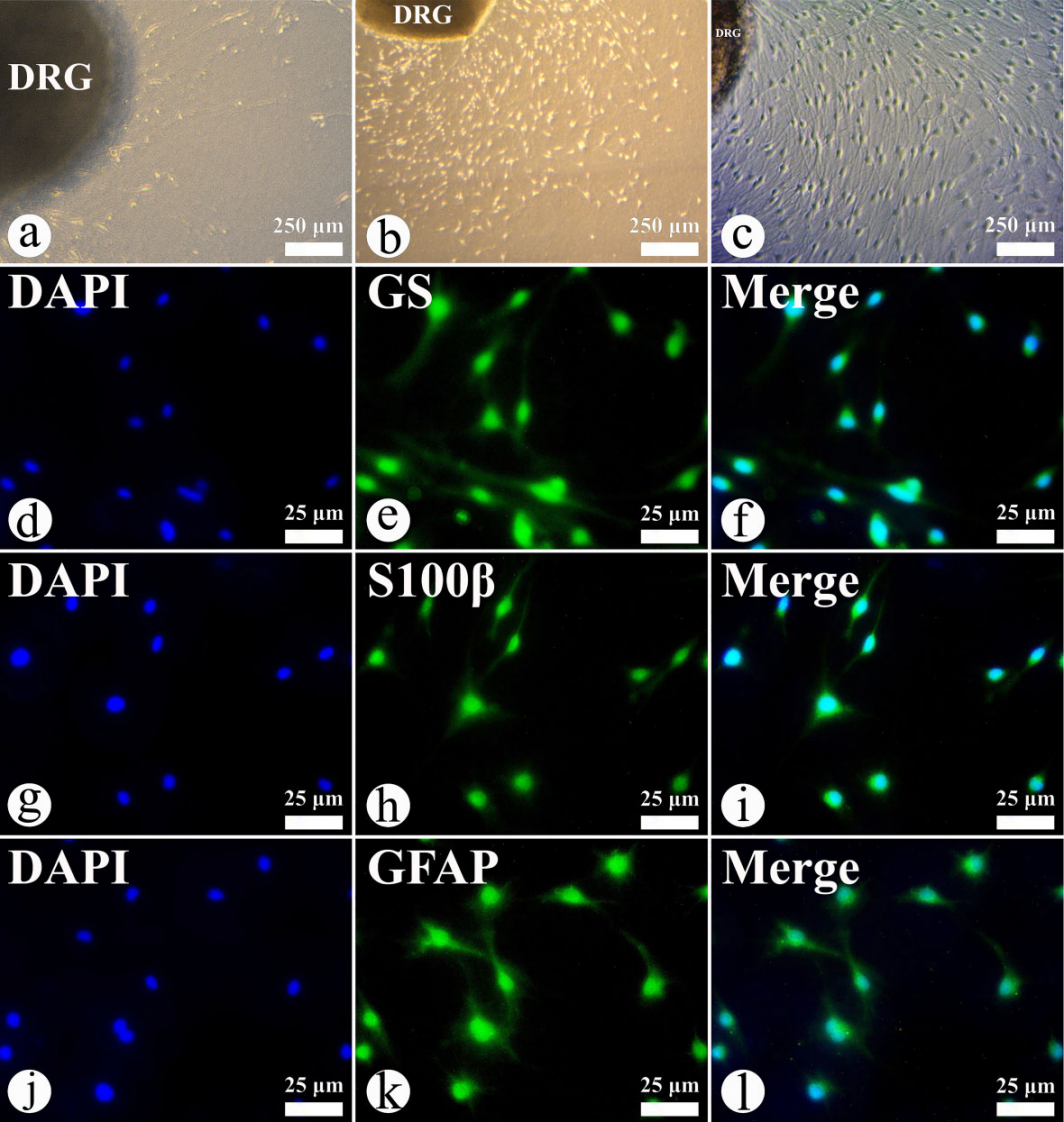
Morphological changes of dorsal root ganglion-derived satellite glial cells (DRG-SGCs) in primary culture. **a**, DRG-SGCs cultured for 1 day, **b**, 7 days, and **c**, 10 days (40×). **d**-**f**, Cells subcultured for 3 days were immunoreactive for glutamine synthetase (GS) (**d**: DAPI, blue; **e**: GS, green; **f**: merged). **g**-**i**: Cells subcultured for 3 days were immunoreactive for S100β (**g**: DAPI, blue; **h**: S100β, green; **i**: merged). **j**-**l**: Cells subcultured for 3 days were immunoreactive for glial fibrillary acidic protein (GFAP) (**j**: DAPI, blue; **k**: GFAP, green; **l**: merged). Scale bars 250 and 25 μm.

The primary culture of DRG from newborn rats was successfully established. A small number of SGCs began migrating from the DRG after one day of culture (Figure 1a), and a significant increase in the migration of SGCs was observed after seven days of culture (Figure 1b). By the tenth day, the proliferating cells were sufficient in number for subculturing (Figure 1c). Immunocytochemical analysis confirmed the identity of the cultured cells as SGCs, with all cells testing positive for the markers GS, S100β, and GFAP (Figure 1d-l).

### The phenotype change of DRG-SGCs was accompanied by alterations in the lnc87717, AKT1, and proBDNF/BNDF signaling pathways

**Figure 2:**
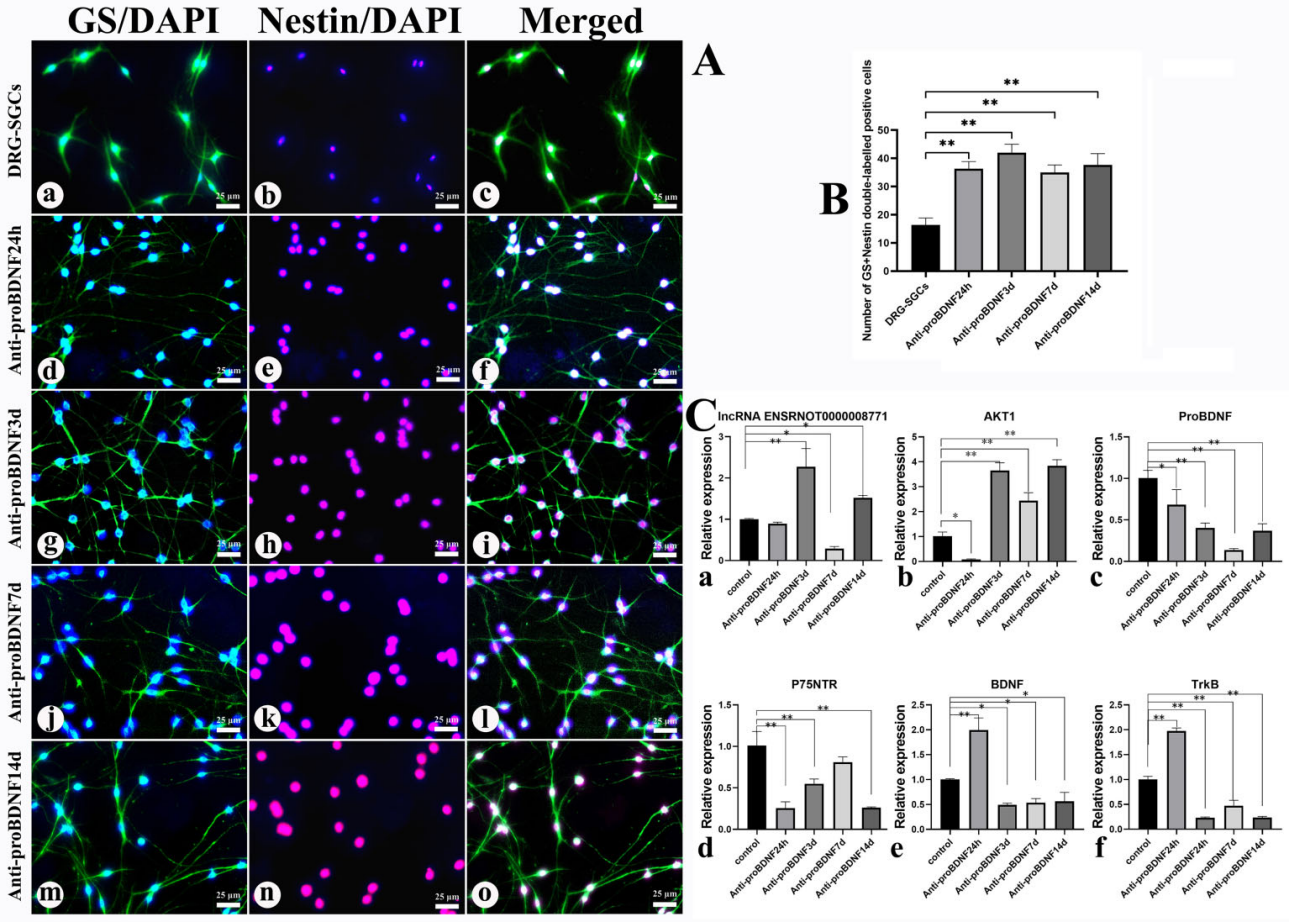
The changes in cell morphology and the expression of lnc87717, AKT1, and proBDNF/BNDF signaling pathways were examined during the differentiation of dorsal root ganglion-derived satellite glial cells (DRG-SGCs). **A**, Immunofluorescence results (**a**-**o**). DAPI staining with blue; nestin staining with red; glutamine synthetase (GS) staining with green. The cells were divided into 5 groups: DRG-SGCs, anti-proBDNF24h, anti-proBDNF3d, anti-proBDNF7d, and anti-proBDNF14d. Scale bar 25 μm. **B**, Number of cells in each group that were double-labeled positive for GS and nestin compared with the DRG-SGCs group. **C**, Expression of **a,** lnc87717, **b**, AKT1, **c**, proBDNF, **d**, p75^NTR^, **e**, BDNF, and **f**, TrkB during the transdifferentiation of DRG-SGCs by qRT-PCR. Data are reported as means and SEM. *P<0.05, **P<0.01 (ANOVA). DRG-SGCs were cultured without anti-proBDNF for 3 days. lnc87717: lncRNA ENSRNOT00000087717.

The cells in the anti-proBDNF induction group exhibited positive immunofluorescence for GS and nestin, with a significant increase in the number of double-labeled positive cells compared to the control group (P<0.05) (Figure 2A and B). Compared with the control group, the expression of lnc87717 was significantly upregulated (P<0.05) in the 3- and 14-day groups induced by anti-proBDNF. In contrast, the expression of lnc87717 was significantly downregulated in the 7-day group compared to the control group (P<0.05). Additionally, the expression of AKT1 was significantly upregulated in the 3-, 7-, and 14-day groups compared to the control group (P<0.01), while it was significantly downregulated in the 24-h group (P<0.05). Furthermore, proBDNF and its receptor p75^NTR^ exhibited significant downregulation at 24 h, as well as on days 3 and 14 after anti-proBDNF induction (P<0.05), with the exception of p75^NTR^ at day 7, which did not exhibit a statistically significant decrease (Figure 2C).

### Expression of AKT1 and proBDNF pathway-related factors in SGCs following downregulation of lnc87717

**Figure 3:**
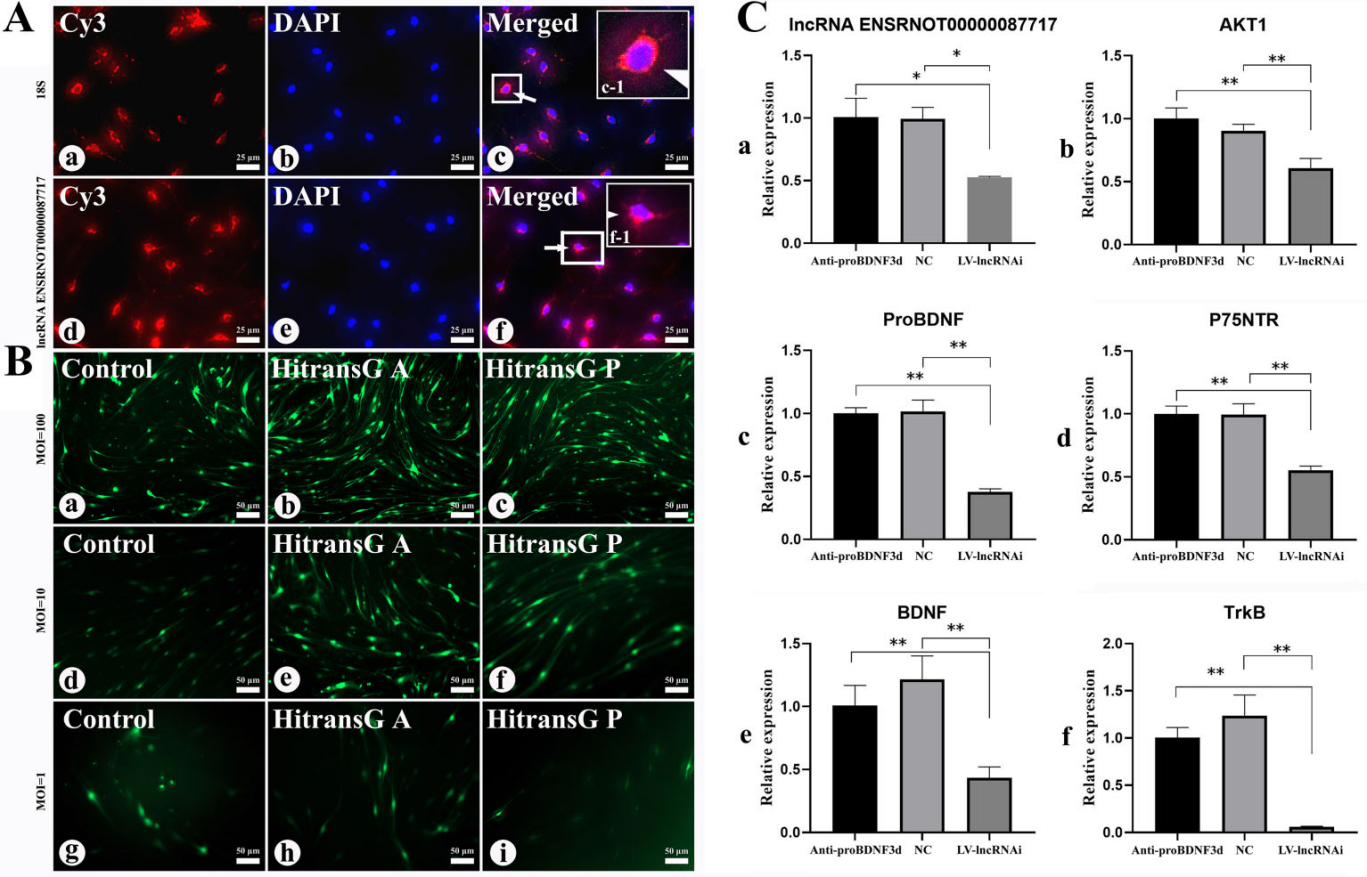
Expression of lnc87717, AKT1, and proBDNF/BNDF signaling pathways-related factors in satellite glial cells (SGCs) was assessed following knockdown of lnc87717. **A**, Localization of lnc87717 in cells, with 18S as the internal reference; red: CY3 labeled by the lncRNA FISH probe; blue: DAPI marks the nucleus (400×). **B**, Lentiviral multiplicity of infection (MOI) assay: GFP-labeled virus-infected dorsal root ganglion-derived satellite glial cells (DRG-SGCs) (green): **a**, **d**, **g**, control group; **b**, **e**, **h**, HiTransG A infection enhancer (25×) group; **c**, **f**, **i**, HiTransG P infection enhancer (25×) group; MOI=100, 10, and 1: MOI=(virus titer×virus volume)/cell number. Scale bars 25 and 50 μm. **C**, qRT-PCR detection of the expressions of lnc87717, AKT1, and proBDNF/BNDF signaling pathways-related factors in SGCs. Data are reported as means and SEM. *P<0.05, **P<0.01 (ANOVA). lnc87717: lncRNA ENSRNOT00000087717.

FISH results indicated that lncRNA87717 was localized in the cytoplasm (Figure 3A). Following knockdown of lnc87717, there was a significant decrease in the expression levels of AKT1, proBDNF, BDNF, TrkB, and p75^NTR^ (P<0.05) (Figure 3B and C).

### The phenotype change of DRG-SGCs was accompanied by alterations in the lnc87717, AKT1, and proBDNF/BNDF signaling pathways following downregulation or upregulation of AKT1

**Figure 4:**
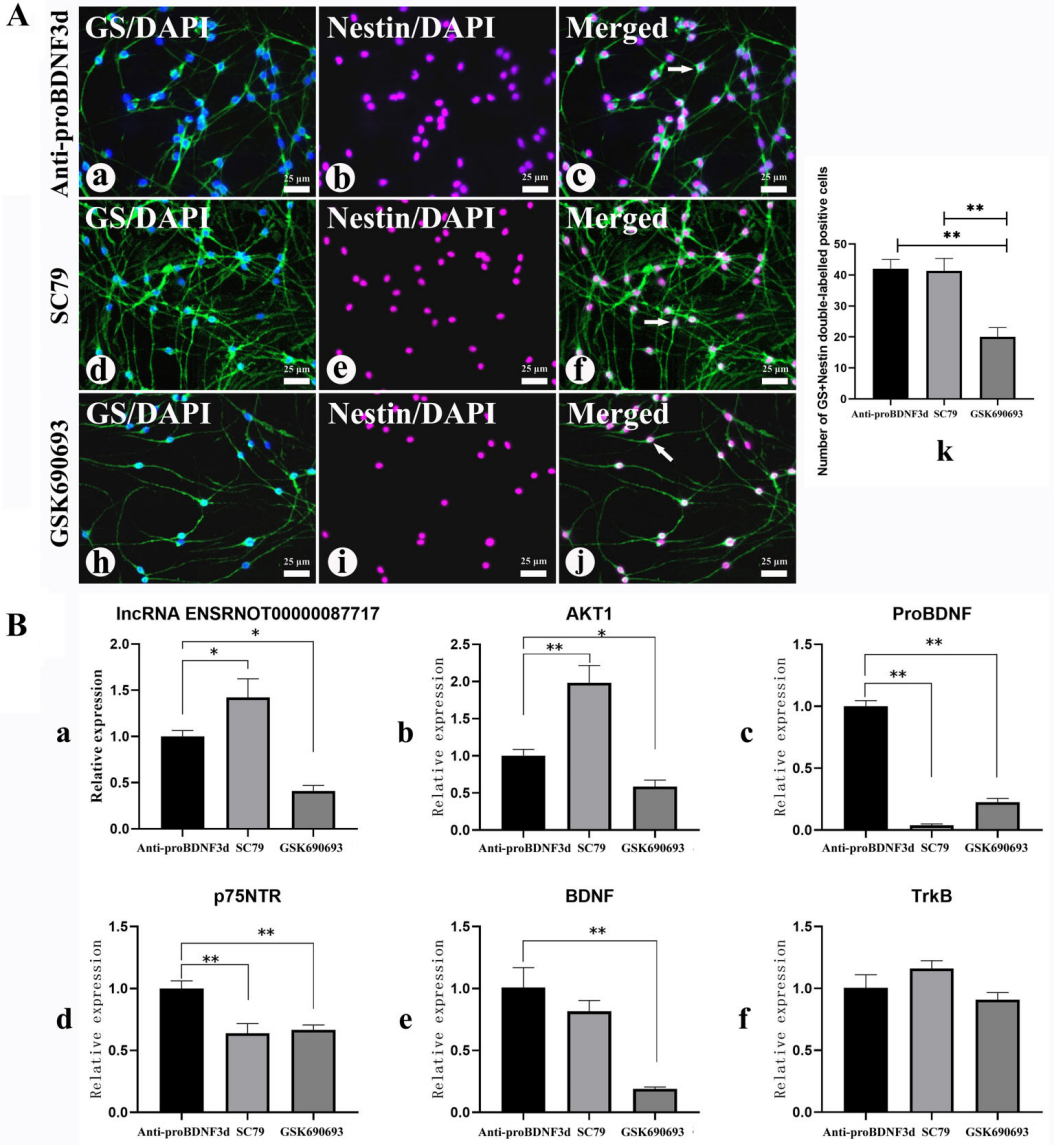
Phenotype change of dorsal root ganglion-derived satellite glial cells (DRG-SGCs) and the expression of lnc87717, AKT1, and proBDNF/BNDF signaling pathways-related factors following AKT1 downregulation or upregulation. **A**, Immunofluorescence detection of nestin and glutamine synthetase (GS) in DRG-SGCs after activation or inhibition of AKT1 in the anti-proBDNF3d, SC79, and GSK690693 groups: DAPI-labeled nucleus (blue) and nestin-labeled neural stem cells (NSCs) (red). GS is a marker for SGCs (green) (400×). **k**, Number of cells in each group double-labeled positive for GS and nestin. **P<0.01. Scale bar 25 μm. **B**, qRT-PCR detection of the expression of lnc87717, AKT1, and proBDNF/BNDF signaling pathways-related factor in SGCs after activation or inhibition of AKT1. Data are reported as means and SEM. *P<0.05, **P<0.01 (ANOVA). lnc87717: lncRNA ENSRNOT00000087717.

The number of nestin-positive SGCs decreased upon inhibition of AKT1 in the control group, whereas an increase in nestin-positive SGCs was observed in the AKT1-activated group (Figure 4A). The expression levels of AKT1 and lnc87717 were significantly upregulated (P<0.05), while the expression of proBDNF and p75^NTR^ was significantly downregulated (P<0.01) in the AKT1-activated group. Furthermore, in the AKT1-inhibited group, there was a significant downregulation in the expression of lnc87717, AKT1, BDNF, TrkB, proBDNF, and p75^NTR^ (P<0.05) (Figure 4B).

### Cell apoptosis and the expression of lnc87717, AKT1, and proBDNF/BNDF signaling pathways in DRGs following downregulation or upregulation of AKT1

**Figure 5:**
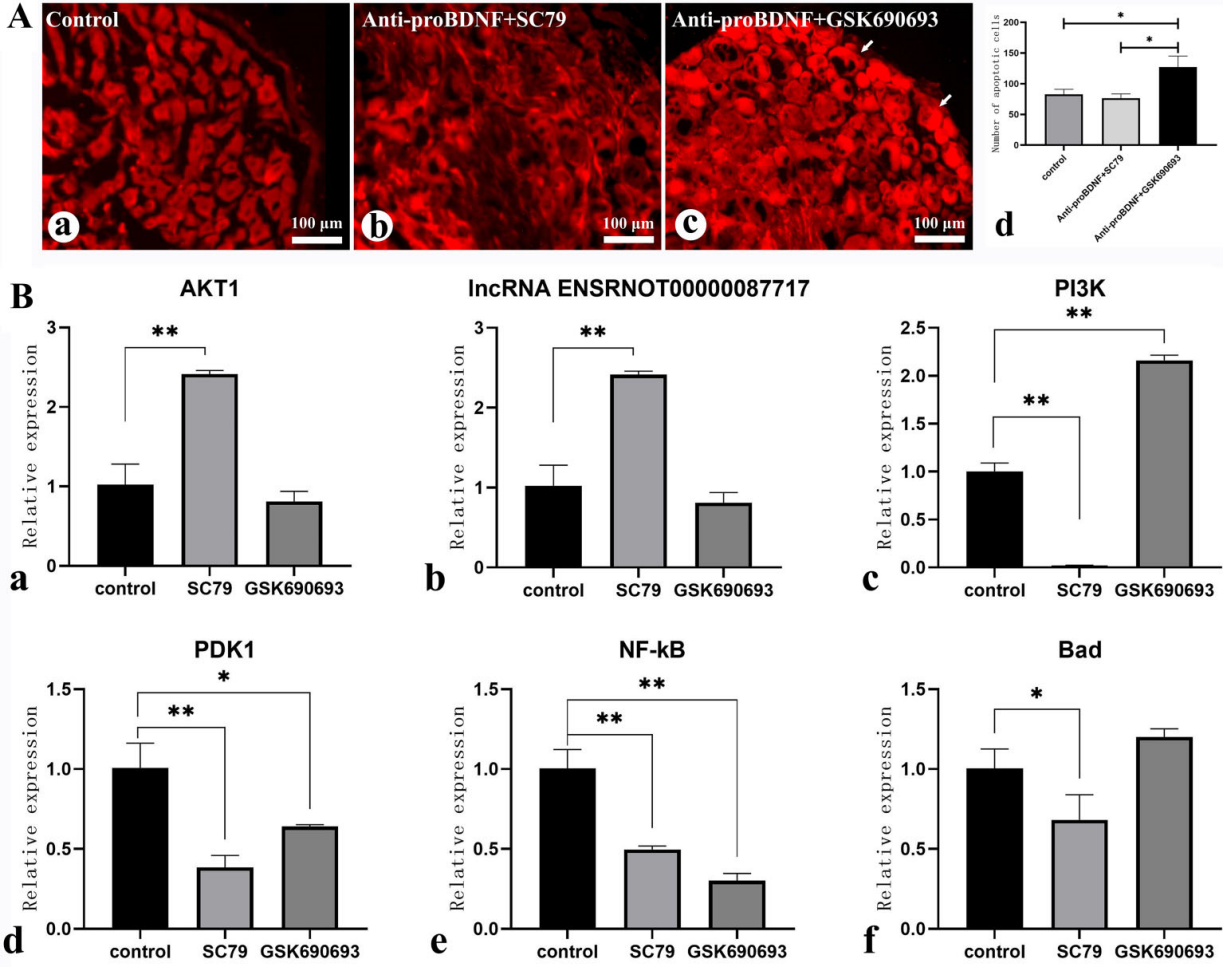
Effects of activation or inhibition of AKT1 on cell apoptosis in the dorsal root ganglion and the expression of AKT1 related genes in rats with sciatic nerve resection. **A**, Apoptosis in DRG was detected by TUNEL. Scale bar 100 μm. **B**, The expression of lnc87717 and AKT1 related factors detected by qRT-PCR in sciatic nerve injury rats with down- or upregulated AKT1 for 3 days. Data are reported as means and SEM. *P<0.05, **P<0.01 (ANOVA). lnc87717: lncRNA ENSRNOT00000087717.

The number of apoptotic cells in the DRG was increased in the AKT1-inhibited group compared to the control group (P<0.05) (Figure 5A). Compared to the control group, the expression of AKT1 and lnc87717 in DRGs was significantly upregulated (P<0.01) in the AKT1-activated group and significantly downregulated in the AKT1-inhibited group. Additionally, the expressions of PI3K, PDK1, NF-κB, and Bad were significantly downregulated (P<0.05) in the AKT1-activated group, whereas the PI3K expression was significantly upregulated in the AKT1-inhibited group (Figure 5B).

## Discussion

Previous studies found that pluripotent stem cells, astrocytes, and neural stem cells can proliferate and differentiate into neurons under certain conditions such as with overexpression of certain transcriptions factors or a combination of small molecules (18-22). Human astrocytes can differentiate into induced pluripotent stem cells using the viral overexpression of four reprogramming factors, Oct4, Sox2, Klf4, and c-Myc (23,24). Previous research has indicated that cultured DRG neurons from neonatal rats can initiate various differentiation pathways of SGC subsets under injury conditions. For instance, SGCs may transdifferentiate into myelinating Schwann cells or oligodendrocytes in the PNS or CNS (25). Our previous investigations have also revealed that subsets of SGCs in adult DRGs possess the ability to undergo differentiation into precursors of sensory neurons during postnatal development and following nerve injury (26,27). It is possible that neurogenesis occurs in the adult rat DRG, as the neuronal count in this region is significantly higher compared to that in neonates. Our previous study (10) found that a subgroup of SGCs in the adult DRG serve as a precursor of sensory neurons during postnatal development and following nerve injury. These cells can differentiate into neurons under the effects of endogenous proBDNF. ProBDNF, precursor in the synthesis of mature BDNF, induces neuronal apoptosis by activating a receptor complex comprising p75^NTR^ and sortilin in cultured sympathetic neurons *in vitro*. (28,29). However, under alterations in the intracellular or extracellular microenvironment, SGCs exhibit the potential to differentiate into neuronal precursor cells and subsequently acquire a neuron-like cellular phenotype.

Our previous study indicates that the differentiation of SGCs involves a complex pattern of lncRNA-mRNA coexpression and lncRNA expression profiling (15). Lnc87717 was significantly upregulated in DRG-SGCs during the differentiation of SGCs. The predicted target gene of lnc87717 was enriched in PI3K/AKT and was identified as AKT1 through the construction of an lncRNA-mRNA interaction network (15). In this study, we observed that the knockdown of lnc87717 inhibited the expression of AKT1. The number of nestin-positive SGCs decreased in the AKT1-inhibition group whereas an increase in nestin-positive SGCs was observed in the AKT1-activated group. This suggests that the differentiation of SGCs is influenced by AKT1. Activation of AKT1 resulted in a decrease in proBDNF expression, accompanied by an increase in the expression of lnc87717 and TrkB. SGCs subjected to AKT1 activation exhibited phenotypic characteristics resembling those of neural stem cells (NSCs), suggesting their potential for differentiation into neuronal precursor cells.

SNI is a specific type of peripheral nerve injury (PNI) that can result in motor and sensory dysfunction, as well as neuropathic pain. Injury to the sciatic nerve induces abnormal motor and sensory functions in the L4-L6 spinal cord segments and the associated bilateral spinal ganglia (30). The limited regenerative capacity of injured neurons represents a significant challenge in the repair of PNI. The transdifferentiation of SGCs into neurons may promote the repair of SNI. In the *in vivo* experiments, lncRNA sequencing was conducted during the transdifferentiation of SGCs. It was predicted that the target gene of lnc87717 is AKT1, which is significantly enriched in the proBDNF signaling pathway. The activation or inhibition of AKT1 can modulate cell apoptosis and the expression of lnc87717, AKT1, and proBDNF/BNDF signaling pathways in DRGs of rats with SNI. AKT proteins regulate a wide variety of cellular functions including metabolism, proliferation, cell survival, growth, and angiogenesis (31-35). AKT acts as a key regulator of the AKT-mTOR signaling pathway, controlling the process of newborn neurons integration in adult neurogenesis. AKT is a downstream protein of the PI3K pathway and transmits signals from phosphatidylinositol PI3K to mediate the effects of growth factors like PDGF, EGF, insulin, and IGF-I (36,37). The AKT pathway also plays a crucial role in the development of neural crest progenitors, stimulating proliferation and survival of peripheral neurons (38). Additionally, AKT can negatively regulate pro-apoptotic proteins through direct phosphorylation, including phosphorylation of the Bcl-2-associated death promoter, leading to its translocation from the mitochondrial membrane to the cytoplasm (39). In this study, by interfering with lnc87717 during the differentiation of SGCs into neurons and modulating AKT1 through its activation or inhibition, we investigated whether the differentiation of SGCs into neurons in the DRG is regulated by lnc87717 and AKT1.

## Conclusion

SGCs in the DRG are multipotent neuronal precursors that exhibit plasticity and can be reprogrammed into neuron-like cells under specific conditions, such as anti-proBDNF induction. This transformation involves the regulation of AKT1 and lnc87717. Investigating the transformation process of SGCs may offer novel insights and experimental bases for studies on development and repair of PNI. While this study elucidated the reprogramming of SGCs into neuron-like cells, further research is required to fully explore the underlying mechanisms.

## Supplementary Material

Click to view [pdf].
