# Supplementary material for: lncRNA ENSRNOT00000087717 mediated differentiation of satellite glial cells derived from dorsal root ganglion via AKT1
Source: Braz J Med Biol Res. 2025 Oct 6;58:e14265. doi: 10.1590/1414-431X2025e14265 (PMC12513695; doi:10.1590/1414-431X2025e14265)
Supplement: Supplementary file 1 [file 1414-431X-bjmbr-58-e14265-suppl.pdf]

Figure S1. Flow diagram of *in vitro* and *in vivo* experiments. SD: Sprague-Dawley; GS: glutamine synthetase.

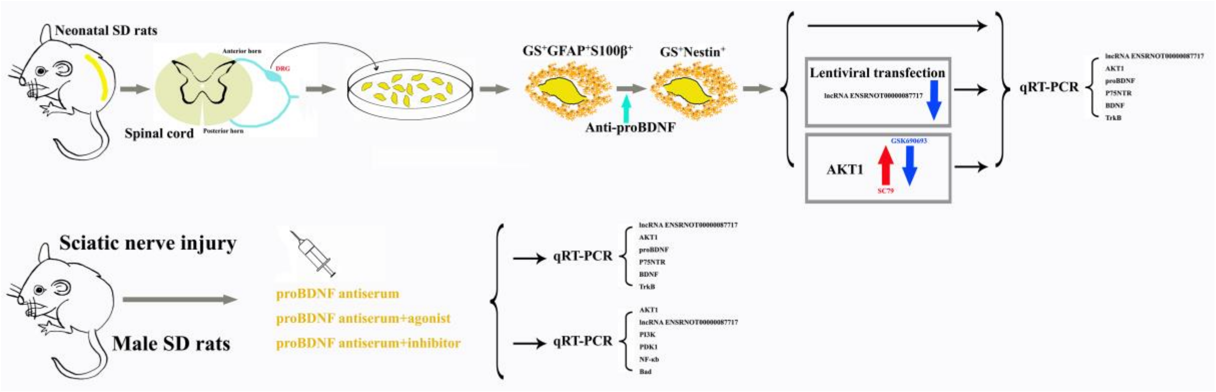

Table S1. Primary and secondary antibodies used in immunocytochemistry.

Primary antibody	Species	Dilution	Company	Corresponding secondary antibody	Species	Dilution	Company
GS	rabbit	1:800	Abcam	Alexa fluor 488	goat-anti-rabbit	1:1000	Abclonal
S100β	rabbit	1:800	Abcam	Alexa fluor 488	goat-anti-rabbit	1:1000	Abclonal
GFAP	rabbit	1:800	Abcam	Alexa fluor 488	goat-anti-rabbit	1:1000	Abclonal
Nestin	mouse	1:800	Abcam	Cy3®	goat-anti-mouse	1:1000	Abclonal

GS: glutamine synthetase; GFAP: glial fibrillary acidic protein; S100β: S-100 protein subunit beta.

Table S2. Primer information.

Gene name	Sequence (5'–3')	MT value
lncRNA ENSRNOT00000087717	Forward: CAGGTCTCCTCCAAGTGTC	57.3
	Reverse: CAGAGTTCTCAACTTGCGG	55.2
AKT1	Forward: ATGAACGACGTAGCCATTGTG	55.6
	Reverse: TTGTAGCCAATAAAGGTGCCAT	54.0
ProBDNF	Forward: ACGGTCACAGTCCTTGAAAAGGT	57.8
	Reverse: ACTGGGTAGTTCGGCACTG	57.3
BDNF	Forward: TCATACTTCGGTTGCATGAAGG	55.8
	Reverse: AGACCTCTCGAACCTGCCC	59.5
TrkB	Forward: CTGGGGCTTATGCTTGCTG	59.5
	Reverse: AGGCTCGGTACACCAAATCCTA	55.8
$p75^{NTR}$	Forward: GTGTGTGAAGAGTGCCCAGA	57.4
	Reverse: CTTCGCATTCAGCATCAGCC	57.4
PI3K	Forward: GTGGCCGTGATGAGTACCTA	55.9
	Reverse: GGCCATTCTTCGTTCCTCAC	56.0
PDK1	Forward: GGGACGGATGCTGTCATCTA	56.0
	Reverse: CGTCATGTCTTTCGGCTCTC	55.8
NF-κB	Forward: GGAGACATCCTTCCGCAAAC	56.0
	Reverse: AGAGATAGCAGTGGGCCATC	55.9
Bad	Forward: GGACAGGCAGCCAATAACAG	55.9
	Reverse: TAAGCTCCTCCTCCATCCCT	55.9

MT: melting temperature.
